# Sex and Age Differences and Psychosocial Determinants of Regular Gambling: Insights from a Community-Based Study

**DOI:** 10.3390/ejihpe15120261

**Published:** 2025-12-18

**Authors:** Claudia Venuleo, Domenico Cuzzola, Tiziana Marinaci

**Affiliations:** 1 Department of Human and Social Sciences, University of Salento, 73100 Lecce, Italy; tiziana.marinaci@unisalento.it; 2 Department of Pathological Addictions, Local Health Authority (ASL) of Lecce, 73100 Lecce, Italy; domcuzz@yahoo.it

**Keywords:** gambling participation, regular gambling, sex and age differences, women, older adults, risk factors, protective factors, public health

## Abstract

Gambling participation among women and older adults has increased, yet research on the psychosocial determinants of gambling in these groups remains limited. This study explored sex- and age-related differences in gambling frequency, the influence of psychosocial factors such as perceived social support, psychological well-being, social connectedness, perceived social approval, and exposure to gambling within one’s network, and how these factors interact with perceptions of the social environment. A community-based sample of 634 adults (69.1% women; 28.7% aged ≥ 60; mean age = 45.7 ± 18.4) completed a survey assessing gambling behaviours and psychosocial variables. Descriptive statistics, bivariate correlations, and binary logistic regression were conducted. Regular gambling was reported by 12% of participants and was significantly more frequent among older adults and men. Higher psychosocial well-being was associated with reduced odds of regular gambling, while being female was also associated with a lower likelihood of regular gambling. Conversely, perceived social approval and an idealized view of the social environment were associated with higher odds of regular gambling, particularly among younger adults. Findings highlight the need for age- and gender-sensitive prevention strategies that strengthen meaningful social connections.

## 1. Introduction

Over the past three decades, public policies liberalizing gambling, along with technological innovations offering new gambling opportunities, have contributed to the global growth of the gambling market (M. W. Abbott, 2020; Research and Markets, 2024). Worldwide, gambling participation—defined as engaging in gambling activities at least once within the last 12 months—ranges from 12% to 92%, with an average prevalence of 55.7% (Dellosa & Browne, 2024).

Although consistent evidence shows that problem gambling disproportionately affects males (Castrén et al., 2018; Gartner et al., 2022) as well as young and middle-aged adults (Dellosa & Browne, 2024), recent research suggests that age and gender differences have narrowed (Gartner et al., 2022; Sharman et al., 2019). Gambling participation among women and older adults has increased in several high-income countries (McCarthy et al., 2019; Volberg, 2003). A recent systematic review and meta-analysis by Tran et al. (2024), based on 166 studies conducted mostly in Western Europe (69), North America (38), and Australasia (33), found that 37.4% of women reported gambling at least once in the past 12 months. Some national surveys report even higher rates. For example, in Scotland, 56% of women reported gambling in the previous year (Scottish Government, 2022); in Finland, 73% of women aged 15–74 had engaged in some form of gambling during the same period (THL, 2023). In Italy—the context of the present study—29.8% of women (approximately 7.9 million aged 18–84) reported having gambled in the past year (Cammarella, 2025). Regarding older adults, while several systematic reviews have addressed the prevalence of problem gambling in this population, comprehensive analyses focusing specifically on gambling participation rates are more limited. The review by Tse et al. (2012) reported that participation rates among older adults varied widely across countries, ranging from 26.6% to 85.6%. More recent national-level studies provide further insights: in Poland, 32.8% of individuals aged 55–64 and 26.8% of those aged 65 and older had gambled in the past 12 months (Lelonek-Kuleta, 2023); in Italy, the participation rate for individuals over 65 is estimated at 25% (Nomisma, 2021).

Despite this growing engagement, empirical evidence on gambling participation and its psychosocial determinants remains limited, as highlighted in the systematic review of psychosocial risk factors for gambling by Nordmyr and Forsman (2020). Gambling research remains largely framed within addiction-centred psychiatric models, in which gambling disorder is conceptualized as the primary concern (Johnson et al., 2023)—a perspective that tends to overlook low-risk gamblers, who constitute the majority of the gambling population, and obscures the influence of social, cultural, interpersonal, and demographic factors, including sex and age, on gambling participation across populations (Kickbusch et al., 2016; Johnson et al., 2023; Marmot, 2005).

Existing literature has frequently identified social isolation, disconnectedness, and the need to engage in socially acceptable recreational activities as key triggers of gambling-related problems (McMillen et al., 2004; Räsänen et al., 2016 Wang & Bellringer, 2022). For instance, Wang and Bellringer (2022) found that lower levels of social connectedness were associated with increased gambling risk, higher psychological distress, reduced engagement in leisure activities, and an overall lower quality of life. The role of unmet social needs in motivating gambling behaviour is also evident in studies showing that both recreational and disordered gamblers may be drawn to gambling for socialization purposes (Nuske et al., 2016), or as a means of coping with the negative emotions associated with social exclusion (Breen & Gainsbury, 2013). Moreover, the risk of problem gambling has been shown to increase in contexts characterized by loneliness and social isolation (Botterill et al., 2016; Elton-Marshall et al., 2018; Floyd et al., 2025). Notably, Floyd et al. (2025) found that social deficits—such as loneliness and frustration resulting from unfulfilled relationships—moderated the relationship between social gambling motives and problem gambling. They argued that whether social motives act as a protective or risk factor depends on the extent to which individuals feel unfulfilled or dissatisfied in their interpersonal relationships. With specific regard to women, a qualitative study by Nuske et al. (2016), based on interviews with both recreational and problem gamblers, found that a key distinction in participants’ narratives concerned their levels of social capital. Problem gamblers reported increased social isolation after major life events, which contributed to their gambling motivation, whereas recreational gamblers described strong social networks as a protective factor. These findings are supported by other research linking social isolation to both the onset and escalation of problem gambling in women (Syvertsen et al., 2023). The importance of social connection has also been highlighted in studies focusing on older adults (for a review, see Sharman et al., 2019). Studies show that older adults often use gambling and visits to gambling venues to foster social ties (Ciofi, 2019; W. Kim, 2020; Lelonek-Kuleta, 2022; Peñalba, 2020; Van der Maas et al., 2019) and reduce isolation (Elton-Marshall et al., 2018; Luo, 2021; McCarthy et al., 2022; Pattinson & Parke, 2016). Significant life changes—such as retirement, bereavement, and declining health—can lead to loneliness and social withdrawal, increasing vulnerability to the appeal of gambling environments (Johnson et al., 2023; McMillen et al., 2004; Venuleo et al., 2021; Zaranek & Lichtenberg, 2008).

The role of socio-cultural norms in shaping gambling involvement has also been acknowledged. For instance, Gainsbury et al. (2014) argued that such norms influence gender roles and shape perceptions of gambling acceptability across age groups, thereby reinforcing demographic patterns. Parental gambling has been linked to both the initiation of gambling (Kalischuk et al., 2006) and the development of problem gambling in adulthood (Ó Ceallaigh et al., 2024; Derevensky & Gupta, 2007; Nower et al., 2022). Perceived approval of gambling within one’s social environment correlates with more favourable attitudes and higher gambling involvement among youth (Delfabbro et al., 2022; Hanss et al., 2014; Marinaci et al., 2021). As Reith and Dobbie (2011) compellingly stated, “It is not enough that gambling opportunities are simply ‘there’: social relationships are the crucial conduit through which they are endowed with meaning and made to matter to the individuals who encounter them” (p. 491). However, there remains a paucity of research specifically examining the impact of having family members and friends who gamble among adults and older individuals.

Beyond interpersonal influences, the way individuals perceive and interpret the broader social environment—its reliability, fairness, and opportunities—also appears to shape gambling behaviour. Research shows that, while non-gamblers or individuals not at risk tend to express greater trust in social norms and institutions and maintain a more hopeful view of the future, those exhibiting problematic gambling behaviour often perceive the social environment as normless and unreliable, characterized by widespread rule-breaking and deep distrust toward institutions such as the government and healthcare (Marinaci et al., 2020; Venuleo et al., 2015, 2016a, 2018). In this context, relying on luck emerges as one of the few perceived strategies for coping with life’s challenges. The perception of the social environment as normless and unreliable has been linked not only to gambling behaviour but also to a range of other risk-related behaviours (De Luca Picione et al., 2023; Venuleo et al., 2022) and broader phenomena such as school dropout (Venuleo et al., 2016a), vaccine hesitancy (Rochira et al., 2019), and public attitudes toward the mafia (Marinaci et al., 2025). These findings suggest that individuals’ views of the social environment may play a moderating role in gambling engagement.

Overall, these findings highlight key areas that warrant greater attention in gambling research and practice.

## 2. The Present Study

The present study was launched at the request of a Local Health Authority in southeast Italy as part of an action-research project titled “L’Azzardo è fare Rete” (“Gambling is Building Networks”), aimed at preventing problem gambling, with a specific—though not exclusive—focus on women aged 18 and older, as well as older adults. Although no universal cutoff defines older gamblers, this study used age ≥ 60 based on the WHO (2018) definition, considering biopsychological changes at this age that may increase vulnerability to both physical and mental conditions often cited as risk factors for gambling participation.

The project aligns with a public health framework that encourages examining how immediate social circles, community ties, and cultural expectations shape behaviours (Dyall, 2004), emphasizing the importance of early intervention and prevention rather than focusing solely on treatment of clinical cases (Delfabbro & King, 2017). Drawing on a sociocultural perspective of hazardous behaviours (Borrell & Boulet, 2005; Marinaci et al., 2021; Messerlian et al., 2005; Oei & Raylu, 2009; Venuleo & Marinaci, 2017; Venuleo et al., 2017), gambling is viewed as a socially meaningful practice that expresses broader aspects of social experience and identity (Crossley, 2001; Rogier et al., 2020).

This article focuses on the research phase of the project, which aimed to explore:

- How gambling habits and frequency varies across socio-demographic groups, with particular attention to differences related to sex and age;
- The role of psychosocial factors—such as perceived social support, psychological well-being, social connectedness (e.g., lower levels of social loneliness), perceived social approval, and exposure to gambling by family and friends—in relation to gambling engagement;
- Whether individuals’ views of the social environment interact with sex and age groups in shaping gambling engagement.

## 3. Materials and Methods

### 3.1. Instruments

A battery of self-report instruments was administered, divided into three sections.

Gambling section

- Gambling habits and frequency. Participants were presented with a list of 10 gambling activities (e.g., slot machines, scratch cards, lottery) and asked whether they had spent money on any of these in the past month (yes/no). If they answered yes to at least one activity, they were then asked to indicate how frequently they had gambled with money during the past month. This one-month reference period served as a behavioural indicator of recent gambling engagement. The rating scale for each type of gambling ranged from 0 = never participated, 1 = once a month, 2 = 2–4 times a month, 3 = 2–3 times a week, 4 = 4–5 times a week, to 5 = 6 or more times a week.
- Problem Gambling. The Problem Gambling Severity Index (PGSI) (Ferris & Wynne, 2001) was used to assess the severity of problematic gambling. The PGSI, part of the Canadian Problem Gambling Index (CPGI), contains 9 items rated on a scale from 0 (never) to 4 (almost always), referring to the past 12 months. The total score ranges from 0 to 27. The PGSI has demonstrated good internal consistency (α = 0.84) and strong criterion-related validity. The Italian validation by Barbaranelli et al. (2013) confirms its internal validity, reliability, and concurrent validity. In this study, the Cronbach’s alpha was 0.90.

Psychosocial factors section

- Perceived approval and exposure to gambling. Two ad hoc indices were developed to assess proximal social norms, based on literature concerning social attitudes and gambling (e.g., Konietzny et al., 2018). Perceived approval was measured using two items: “My friends approve of gambling” and “My family members approve of gambling.” Exposure to gambling was assessed with two items: “Some of my friends gamble” and “Some of my family members gamble.” Each item was rated on a four-point Likert scale (1 = strongly disagree to 4 = strongly agree), with higher scores indicating greater approval or exposure. Given the brevity of the scales, reliability was evaluated using the Spearman–Brown coefficient, which is appropriate for two-item measures (Eisinga et al., 2013). The coefficients were ρ = 0.44 for perceived approval and ρ = 0.41 for exposure, indicating adequate internal consistency for these short scales.
- Perceived Social Support. The Poortinga’s Scale of Perceived Social Support (Poortinga, 2006) was used to evaluate the availability of instrumental and emotional support in everyday life. The instrument consists of seven statements (e.g., “There are people I know—among family or friends—who: do things to make me happy; make me feel loved; are reliable in any circumstance; would make sure I’m taken care of if I needed it; accept me as I am; make me feel like an important part of their life; give me support and encouragement”). Participants responded using a 3-point scale: 1 = “Not true,” 2 = “Partly true,” and 3 = “Definitely true.” Higher scores indicate greater perceived social support. The instrument demonstrated good reliability (α = 0.88) in previous research (Marinaci et al., 2021). In this study, the Cronbach’s alpha was 0.91.
- Loneliness. Perceived loneliness was assessed using the De Jong Gierveld Loneliness Scale (DJGLS; De Jong Gierveld & Kamphuls, 1985). This scale includes 11 items measuring perceived loneliness and comprises two subscales: one reflecting a lower-than-desired number of social relationships (social loneliness) and another reflecting a lack of desired intimacy in close relationships (emotional loneliness) (De Jong Gierveld & van Tilburg, 2010). Sample items include “I miss having a really close friend” (emotional loneliness subscale) and “There are many people I can count on when I have problems” (social loneliness subscale). The scale uses a 5-point Likert scale ranging from 0 (never) to 5 (always). The DJGLS can be used either as a global measure of loneliness or to assess emotional and social loneliness separately. It has demonstrated adequate internal consistency, with a Cronbach’s alpha of 0.86, and has been validated for construct validity. In this study, the alpha value was 0.81.
- Well-being. The Italian version of the Flourishing Scale (FS) (Di Fabio, 2016; Diener et al., 2010) was used to measure well-being. The FS provides an overview of individuals’ perceived social and psychological functioning. It consists of 8 items rated on a 7-point Likert scale (1 = strongly disagree to 7 = strongly agree), with total scores ranging from 8 to 56. Higher scores indicate greater psychological strengths. The Italian version demonstrated good internal consistency, homogeneity, and validity (Giuntoli et al., 2017). In this study, the Cronbach’s alpha was 0.92.
- View of the social environment. The View of Context (VOC) questionnaire (Ciavolino et al., 2017) is a self-report instrument designed to map the cultural models through which individuals interpret their social context (Mossi & Salvatore, 2011). It assesses perceptions and evaluations of both micro- and macro-social contexts, such as the evaluation of the place of residence, perceived reliability of public institutions (e.g., police, hospitals, and schools), and endorsement of moral and civic values (e.g., rule compliance and sharing). The questionnaire comprises 26 items, rated on four-point Likert scales (e.g., “Not at all” to “A lot” or “Very unreliable” to “Very reliable”). The instrument has demonstrated satisfactory construct validity (Ciavolino et al., 2017) and has been used in several studies to explore links between worldviews, psychosocial malaise, and maladaptive behaviours such as problematic gambling, internet use, and substance consumption (Ciavolino et al., 2017; Ferrante et al., 2022; Marinaci et al., 2021; Venuleo et al., 2016b). In this study the alpha value is 0.67.

Respondent’s Socio-Demographic Characteristics.

At the end of the questionnaire, participants were asked to specify their sex, age, educational level and perceived income level.

### 3.2. Recruitment Procedure

The project identified local services and associations predominantly attended by women or older adults, along with parishes and various cultural and recreational groups that, in our area, attract both men and women across a wide age spectrum. These included organizations focused on promoting and developing the local community, initiatives for social promotion and civic engagement, and services supporting senior citizens or the welfare of older adults. Together, these setting were considered key contexts for identifying individuals at risk of gambling, detecting psychosocial distress, and promoting well-being. A letter explaining the purpose of the project was sent to 146 local organizations, along with an invitation to attend a presentation event about the research-intervention project.

Fifty-one organizations accepted the invitation and agreed to become partners and active promoters of the planned research-intervention activities. Within these participating organizations, specific meetings were held with beneficiaries, all aged 18 or older, to invite them to complete the questionnaire. The survey was administered both online (via Microsoft Forms) and in paper-and-pencil format to facilitate participation among older adults; in-person assistance was provided by a trained university student when needed.

Two members of the research team introduced the study and its aims: to investigate participants’ gambling habits and to explore their perceptions of the quality of their relationships and social networks. The voluntary nature of participation and the anonymity of responses were emphasized, and participants were encouraged to ask questions or seek clarification as needed. Participants were informed that data would be analyzed in aggregate form and that only the research team would have access to the data. Written informed consent was obtained from all participants. No incentives were provided. On average, participants spent approximately 20 min completing the survey.

### 3.3. Participants

A total of 634 participants (mean age = 45.7 years, SD = 18.4; 69.1% women; 28.7% aged over 60) completed the survey. The socio-demographic characteristics of the sample are presented in Table 1.

### 3.4. Data Analysis

#### 3.4.1. Descriptive Statistics

Gambling habits, gambling frequency, and problem gambling rates were examined in the total sample and further analyzed by sex and age group (<60 vs. ≥60 years). To explore gambling habits, a frequency analysis was conducted by grouping gambling activities into four categories based on modality and setting, in line with classifications commonly used in gambling research (Fiedor et al., 2025): (1) lotteries (e.g., scratch cards, Lotto, 10eLotto); (2) slot machines and bingo; (3) betting (i.e., sports betting, race betting, private betting), and (4) card games (blackjack and poker). Regarding gambling frequency, participants were classified according to the frequency of gambling they reported over the past month. They were then grouped into three categories: non-gamblers, occasional gamblers—those who reported gambling once during the past month—and regular gamblers—those who reported gambling two or more times during the past month.

Regarding problem gambling rates, following indications from Ferris and Wynne (2001), subjects scoring 0 on PGSI were classified as “non-problem” gamblers, those scoring between 1 and 2 were classified as “low risk” gamblers, those scoring between 3 and 7 as “moderate-risk” gamblers and those scoring higher than 7 as “problem” gamblers. Chi-square procedures and post hoc analysis of adjusted standardized residuals were used to explore significant differences related to sex and age.

Means and standard deviations were computed for all psychosocial variables, both in the total sample and disaggregated by sex and age group (<60 or ≥60). As the Shapiro–Wilk test indicated significant deviations from normality (all *p* < 0.05), robust statistical procedures were applied in subsequent analyses.

#### 3.4.2. Preliminary Analysis

A preliminary analysis was conducted on the View of the Context Questionnaire (VOC). The methodology underlying the VOC requires applying Multiple Correspondence Analysis (MCA) (Lebart, 1998) to participants’ responses in order to identify factorial dimensions that summarize the relationships between nominal or ordinal variables within the specific sample (Ciavolino et al., 2017). In line with prior applications of the VOC (Ciavolino et al., 2017; Ferrante et al., 2022; Marinaci et al., 2021; Kerušauskaitė et al., 2023), these dimensions were interpreted as Latent Dimensions of Sense (LDSs)—that is, affect-laden, oppositional structures that organize individuals’ interpretations of their social environment.

In this study, the first dimension contrasts two patterns of responses interpreted as distinct models of engagement with the social environment: radical distrust (−) versus moderate trust (+) (see Appendix A: Table A1). The radical distrust pole (−) encompasses items with extreme responses on Likert scales (e.g., “strongly agree,” “not at all”) and negative content (e.g., public services and institutions are unavailable, the future is unpredictable, people cannot change, or one cannot count on others). An anomic view emerges, characterized by the perception that the only prevailing rule is to act without scruples and to side with the most powerful. Conversely, the moderate trust pole (+) is characterized by moderate Likert responses (e.g., “quite agree,” “quite disagree”) associated with a view of institutions and people as somewhat reliable, a cautiously optimistic outlook on the future, and a belief in one’s ability to effect change.

The second dimension contrasts two response patterns interpreted as differing ways of evaluating the social environment: moderate criticism (−) versus idealization (+). This dimension reflects a general tendency to either express nuanced, measured judgments or to provide globally positive evaluations of objects and situations (see Appendix A: Table A2). The moderate criticism pole (−) aggregates responses located at intermediate points on the Likert scales (e.g., “not very,” “somewhat”). The overall content tends to be negative: institutions and services are perceived as somewhat untrustworthy, and there is moderate agreement with statements expressing anomic logic (e.g., “To succeed in life, it is important to have few scruples and to side with the strong”). In contrast, the idealization pole (+) includes responses associated with the extreme ends of the Likert scales (e.g., “strongly disagree,” “very reliable,” “much better”), and reflects a consistently positive view of the social environment. Participants at this end express strong trust in people and institutions, optimism about the future, and strong disagreement with statements reflecting anomic beliefs. The context is thus portrayed as a pleasant, functional, and trustworthy space.

After applying the inertia adjustment formula (Benzécri, 1979), it was found that the first factorial dimension of the VOC (VOC1) accounted for 39.9% of the explained variance, while the second dimension (VOC2) accounted for 29.7%, resulting in a total of 69.6% of the inertia explained by the two dimensions.

The MCA provides a measure of the association between each respondent and the factorial dimensions, expressed as their coordinates on each dimension. The more closely a respondent’s pattern of answers aligns with the profile characterizing a given dimension, the higher their score (coordinate) on that dimension. Thus, each respondent’s view of the social environment is captured through two continuous variables—VOC1 and VOC2—which were used in subsequent analyses.

To evaluate whether the three components of the Psychosocial Well-being Index captured complementary rather than redundant aspects of adjustment, their intercorrelations were examined. The associations were small to moderate, confirming that the subcomponents contributed distinct information and supporting their aggregation into a single composite index (see Appendix A: Table A3). Based on theoretical considerations and the empirical distinctiveness of its components, the Psychosocial Well-being Index was computed as the standardized mean of the three dimensions. Its association with gambling frequency was small but statistically significant (Appendix A: Table A3). The effect size (r = −0.14, *p* < 0.001) is consistent with values commonly reported in community-based, non-clinical samples of gamblers (e.g., Moravec et al., 2025; Oksanen et al., 2021; Palomäki et al., 2025). Given its theoretical coherence and empirical distinctiveness from the other psychosocial variables, the index was retained for subsequent analyses.

Finally, non-parametric Mann–Whitney U tests were used to compare psychosocial variables across sex and age groups. Differences between men and women, and between younger (<60 years) and older adults (≥60 years), were examined for all psychosocial indicators.

#### 3.4.3. The Prediction of Gambling Engagement

A binary logistic regression model was used to estimate the likelihood of regular gambling, defined as gambling two or more times during the past month based on participants’ self-reported gambling frequency. This operationalization ensured that the outcome reflected recent participation rather than sporadic or annual gambling.

The model included the following predictors: psychosocial well-being (the index computed as the standardized mean of perceived social support, psychological well-being, and reversed social loneliness), perceived approval of gambling, exposure to gambling within the social environment, and the two factorial dimensions of the View of the Social Environment (VOC1 and VOC2). Interaction terms were incorporated to explore whether sex and age moderated the relationships between VOC1, VOC2, and regular gambling engagement. Model fit was evaluated using the Akaike Information Criterion (AIC) and McFadden’s pseudo-R^2^. Results are presented as odds ratios (OR) with 95% confidence intervals (CI). All analyses were conducted using R (version 4.5.1) and SPAD (version 5.5).

## 4. Results

### 4.1. Gambling Habits, Frequency and Problem Rates

As shown in Table 2, the most common gambling activities were lotteries (e.g., scratch cards, Lotto, 10eLotto), with 56.5% of respondents reporting participation, followed by betting (20.7%), slot machines and bingo (9.0%), and card games such as blackjack and online poker (2.8%). Chi-square analyses revealed a significant association between sex and betting (χ^2^ = 53.63, *p* < 0.001), with men (57.3%) participating more and women (42.7%) less than expected (|residual| ≥ 1.96). No significant sex differences were found for lotteries, slot machines/bingo, or card games. Similarly, no significant age differences were observed across gambling categories, indicating comparable participation patterns between younger (<60 years) and older (≥60 years) respondents.

With respect to gambling frequency, 70.8% of participants were classified as non-gamblers, 17.2% as occasional gamblers (once a month), and 12.0% as regular gamblers (more than once a month). As shown in Table 2, gambling frequency differed significantly by sex (χ^2^ = 73.30, *p* < 0.001), with women more often no gambling (78.5%) and men more often regular gamblers (28.1%) (|residual| ≥ 1.96). Significant differences also emerged by age group (χ^2^ = 11.77, *p* < 0.005), with younger participants more frequently non-gamblers (73.9%) and older adults more often regular gambling (18.7%) (|residual| ≥ 1.96).

Regarding gambling severity, most respondents were classified as no-problem gamblers, while low-risk, moderate-risk, and problem gamblers were comparatively less frequent (Table 2). Sex differences were significant (χ^2^ = 46.01, *p* < 0.001), with women more often non-gamblers (93.2%) and men more often in all risk categories beyond non-gambling (low-risk 13.3%, moderate-risk 8.7%, problem 4.1%) (|residual| ≥ 1.96). Age differences were also observed (χ^2^ = 9.59, *p* < 0.05), with problem gambling relatively more prevalent among older participants (3.8%) (|residual| ≥ 1.96).

### 4.2. Distribution of Psychosocial Variables

As shown in Table 3, non-parametric Mann–Whitney tests revealed significant differences in psychosocial variables across sex and age groups. Compared to men, women reported lower perceived approval of and exposure to gambling within their family and friendship networks, higher psychosocial well-being, and a greater tendency to express a trusting view of the social environment (positive polarity of VOC1). No significant sex differences were found for the second contextual dimension (VOC2). Regarding age, participants aged 60 and over, compared to younger participants, reported lower psychosocial well-being, lower perceived approval of and exposure to gambling, and a greater likelihood of expressing a deeply distrustful view of the social environment (negative polarity of VOC1). No significant age differences were observed for VOC2 (ways of evaluating the social context).

### 4.3. Binary Logistic Regression Model

The binary logistic regression model revealed several significant associations (Table 4). The Psychosocial Well-being Index showed a small but statistically significant negative association with gambling frequency (OR = 0.71, 95% CI [0.54, 0.94], *p* < 0.05). In contrast, perceived approval of gambling substantially increased the odds of regular gambling engagement (OR = 1.58, 95% CI [1.23, 2.04], *p* < 0.001). Regarding the view of the social environment, an idealizing perspective (positive pole of VOC2) emerged as being strongly associated with regular gambling (OR = 2.94, 95% CI [1.31, 6.49], *p* < 0.01), whereas the trust versus distrust dimension (VOC1) did not reach statistical significance. A significant interaction between VOC2 and age was also found (OR = 0.98, 95% CI [0.96, 0.99], *p* < 0.05), indicating that the risk-enhancing effect of idealization was stronger among younger adults and diminished with increasing age. Overall, the model demonstrated satisfactory explanatory power given the sample composition (McFadden’s R^2^ = 0.24; AIC = 373.19).

## 5. Discussion

The main objectives of this study were: (a) to explore sex and age differences in gambling habits and frequency and (b) to examine the role of psychosocial and cultural factors in either preventing or promoting regular gambling. The following sections first discuss the findings related to gambling habits and frequency. Subsequently, we provide an overview of the distribution of key variables by sex and age, followed by an analysis of the protective and risk factors for regular gambling identified in the regression model. This includes a detailed discussion of each psychosocial and cultural variable considered. Finally, the role of sex and age is examined.

### 5.1. Gambling Habits

Lotteries emerged as the most reported form of gambling, consistent with previous research showing that they are generally perceived as low-risk, socially acceptable, and accessible—particularly in offline, retail-based formats (Granero et al., 2023). Interestingly, significant sex differences were observed only in relation to betting, with men more likely to engage in this form of gambling. This aligns with studies suggesting that men are more attracted to skill-based or competitive forms of gambling such as sports betting, often motivated by excitement or perceived expertise (McCarthy et al., 2019; Theodorou et al., 2025). In contrast, no sex differences were found for lottery or slot machine participation. This pattern may reflect the widespread normalization of these activities across demographic groups, particularly in regions where these products are heavily marketed and widely available (Edet, 2025), as observed in Italy (Lenzi Grillini et al., 2025), which is the context of the present study. This, in turn, may reduce the gender-based stigma to which women are often more exposed, in Italy as in other European countries (Baxter et al., 2016; Collard et al., 2022; McCormack et al., 2014; Rolando et al., 2023), making them equally likely to participate. The absence of significant age-related differences across gambling activities may reflect the widespread accessibility and cultural normalization of certain gambling products—particularly lotteries—which remain highly prevalent across age groups. In Italy, for instance, products such as scratch cards and national lotteries are commonly available in everyday retail settings, reducing the technological or mobility barriers that might otherwise deter older adults. Moreover, previous research has noted that older adults often prefer games of chance with low perceived risk and familiar formats (Subramaniam et al., 2015), which may explain their comparable participation in lotteries or bingo.

### 5.2. Gambling Frequency

While non-gamblers represented the majority of the sample, the proportion of individuals who reported regular gambling (12%)—closely aligned with the percentage classified as at-risk gamblers (12.7%)—warrants particular attention. This is especially notable given that the recruitment strategy did not target gambling-specific venues (e.g., bingo halls or betting centres), but rather everyday community settings such as parishes, cultural centres, and recreational facilities. Prior research has consistently shown that the risk of gambling-related harm increases progressively with gambling frequency (Currie et al., 2006; Young et al., 2024). Additionally, several studies have highlighted that women tend to initiate gambling later in life but may progress more rapidly to problematic gambling—a phenomenon referred to as the “telescoping effect” (e.g., Blanco et al., 2006; Grant et al., 2012). From both a public health and population-level perspective, understanding the factors that influence the transition from recreational to problematic gambling remains a key priority (Messerlian et al., 2005; Reith et al., 2019). The findings of the current study underscore the importance of considering both individuals’ interpersonal relationships and their broader perceptions of the social environment in understanding and addressing gambling behaviours.

### 5.3. Sex and Age Differences in Psychosocial and Cultural Variables

Women, compared to men, were overrepresented among non-gamblers and reported lower levels of perceived approval of gambling within their social networks, lower exposure to gambling, higher psychosocial well-being, and a greater tendency to express a trusting view of the social environment. In contrast, individuals aged 60 and over were overrepresented among regular gamblers and, despite lower approval and exposure, reported lower psychosocial well-being and a more distrustful view of the social environment—previously identified as a risk factor for problem gambling (Marinaci et al., 2020; Venuleo et al., 2016b).

These sex- and age-related differences in social capital are consistent with existing literature. Prior research has shown that women typically possess stronger and more supportive social networks (K. Kim et al., 2020), are more inclined to seek help from others—facilitating adaptive coping strategies—and report higher levels of perceived social support (Gul et al., 2018; Kneavel, 2021; Melchiorre et al., 2013). However, cross-national differences are also observed depending on country-level gender-role attitudes. For example, Cohn-Schwartz and Schmitz (2024), examining gender differences in social relationships across 15 European countries, including Italy, found that in contexts with more egalitarian gender-role attitudes, women had larger social networks, whereas in countries with more traditional gender-role attitudes, such as Italy, networks were smaller but closer and more family-oriented. Social support has been widely recognized as a protective factor that can reduce the risk of developing gambling problems, even in the presence of other vulnerabilities (Barone & Graffigna, 2025). The current findings further support this protective role, with women less likely to gamble regularly and underrepresented in at-risk groups. The age-related differences in social capital observed here are also aligned with previous studies (Antonucci et al., 2004; Bowling, 2011; Brajša-Žganec et al., 2018). For instance, Bowling (2011) found that individuals over 65 reported having significantly fewer people to rely on for emotional or practical support during times of crisis, compared to younger adults. However, the literature is not entirely conclusive. Evidence suggests that perceived social support in later life is shaped by a complex interplay of factors, including cultural norms, physical health, social and economic policies and the availability and structure of social networks (Calcagnini & Perugini, 2019; Melchiorre et al., 2024; Moore et al., 2015). For instance, Calcagnini and Perugini (2019) highlight an unequal distribution of social capital at the provincial level in Italy, with Northern provinces endowed with higher levels of social capital than Southern provinces, such as those focused on in our study, which are typically characterized by limited economic resources and infrastructure. Moreover, the effectiveness of social support from family and friends may vary across the lifespan (Nakash et al., 2022), potentially influencing its role in protecting against maladaptive behaviours such as problematic gambling.

### 5.4. Protective and Risk Factors of Regular Gambling

A key finding is that the Psychosocial Well-Being Index—combining perceived social support, psychological well-being, and reverse-coded loneliness—was more strongly associated with gambling frequency than any individual component. This supports the idea that these factors have complementary, rather than overlapping, relationships with gambling behaviour. The result aligns with multifactorial models of risk behaviour (Griffiths, 2005), highlighting how vulnerability to gambling is shaped by the interplay between personal and social resources.

The regression model indicated that a one-unit increase in the Psychosocial Well-Being Index was associated with a 29% reduction in the likelihood of regular gambling in the total sample (OR = 0.71). The simultaneous presence of higher subjective well-being—potentially reflecting greater internal resilience—and social capital (i.e., strong social support and social connectedness) may buffer the psychological impact of critical life events, facilitate access to adaptive coping resources, and reduce the likelihood of engaging in gambling as a compensatory or escapist strategy (Oksanen et al., 2018). This interpretation aligns with findings from community studies which indicate that reduced social support, loneliness and lower well-being may heighten vulnerability to gambling involvement (Botterill et al., 2016; Oksanen et al., 2021). However, the directionality of this association should be interpreted with caution. Qualitative evidence further suggests that gambling participation, even at non-problematic levels, can progressively weaken social connectedness by limiting opportunities for meaningful engagement in everyday relational contexts (Nuske et al., 2016; Syvertsen et al., 2023). This potential for reciprocal rather than unidirectional processes underscores the need for longitudinal research to clarify how psychosocial vulnerabilities and gambling behaviours mutually influence each other and evolve over time.

Perceived approval of gambling from family and friends was associated with a 58% increase in the likelihood of regular gambling (OR = 1.58), whereas merely having family members or friends who gamble was not significantly related to gambling frequency. This discrepancy aligns with Social Learning Theory (Bandura, 1977), which suggests that behavioural modelling is more effective when the behaviour is socially reinforced or approved, rather than merely observed. It is also consistent with the Theory of Planned Behaviour (Ajzen, 1991), which highlights perceived approval—rather than others’ behaviour per se—as the key component of subjective norms shaping behavioural intentions. Previous studies among college students have shown that perceived social approval (i.e., injunctive norms), reflecting the belief that significant others endorse or support gambling, is associated with both gambling frequency and severity (Delfabbro et al., 2022; Neighbors et al., 2002). In contrast, the presence of gambling behaviour among peers or family members—without explicit approval—does not consistently predict gambling engagement (Neighbors et al., 2006). Our findings support the idea that explicit social approval provides a stronger normative cue than passive exposure to gambling behaviour, even in adult populations. However, even in this case, the directionality of this association should be interpreted with caution. It is equally possible that engagement in gambling encourages affiliation with social circles in which gambling is more positively regarded or fosters the development of social networks composed of other gamblers, which in turn may lead to higher perceived social approval (Russell et al., 2018).

Holding an idealized view of the social environment was associated with almost threefold higher odds of regular gambling in this sample (OR = 3.68). While this finding may not be immediately intuitive, it is theoretically plausible. An idealized perception—likely disconnected from the more critical or problematic aspects of reality—may contribute to the normalization of gambling behaviour and the underestimation of its potential risks. This may be particularly relevant among younger individuals, whose critical understanding of social and normative dynamics is still developing. It is worth noting that the idealized view is characterized by homogeneously and strongly positive evaluations of the social environment, often reflected in extreme responses on the Likert scale. Such evaluative patterns can be interpreted as markers of heightened emotional activation, which may impair one’s capacity to regulate behaviour (Salvatore & Freda, 2011). Previous research has shown that increased affective activation—associated with a diminished ability to use reality as a regulatory framework for thoughts, desires, and beliefs—raises the likelihood of engaging in risk behaviours (Ferrante et al., 2022; Venuleo et al., 2015), including problem gambling (Marinaci et al., 2020). Other studies have emphasized the link between emotional complexity—understood as the ability to articulate nuanced and differentiated meanings across various experiential domains—and greater interpersonal adaptability (Kang et al., 2003; Kang & Shaver, 2004; Venuleo et al., 2020).

The lack of a significant association between a distrustful view and gambling frequency is an unexpected finding. Previous studies have found that respondents who tend to express negative and highly polarized evaluations of their micro- and macro-social environments (captured by the “radical distrust” pole of VOC1) are more likely to engage in problem gambling (Marinaci et al., 2020; Venuleo et al., 2016b). However, our study focused on regular (non-problematic) gambling, which may be driven by different underlying motivations and psychosocial dynamics. The absence of a significant link in our findings suggests that, while radical distrust may be associated with more severe, dysfunctional patterns of gambling, it may not play the same role in predicting more moderate or socially integrated forms of gambling behaviour. In this sense, radical distrust might act more as a risk factor for escalation rather than as a direct driver of gambling frequency. Regular gambling –particularly when not accompanied by loss of control or negative consequences—may be driven more by recreational or social factors than by psychological and emotional states related to distrust. This interpretation highlights the importance of considering both the severity and the psychosocial context when examining gambling behaviours. Future longitudinal or comparative studies are needed to clarify how trust and distrust interact with different forms and levels of gambling involvement, and to explore whether radical distrust indeed plays a more prominent role in the escalation toward problematic gambling compared to regular, socially integrated gambling.

### 5.5. The Role of Sex and Age in Regular Gambling

Women are 84% less likely to engage in regular gambling compared to men (OR = 0.16). Given the cross-sectional nature of the study, this association does not imply causality but may reflect underlying psychosocial and cultural differences between sexes. Research has shown that men are generally more likely to gamble for reasons such as excitement, competition, or the desire to win, whereas women are more often motivated by the need to cope with negative emotional states such as anxiety, stress, or loneliness (Blanco et al., 2006). These motivational differences may reflect broader patterns of emotional regulation and coping strategies, which in turn may influence the frequency and intensity of gambling. Women also tend to exhibit lower risk-taking and greater sensitivity to the negative consequences of gambling (Potenza et al., 2001). Evidence from Italian population studies supports this interpretation. Research consistently shows that Italian men display higher gambling participation, greater acceptance of betting, and a stronger orientation toward competitive or strategic games, whereas women tend to prefer low-stakes traditional formats such as lotteries or bingo (Bastiani et al., 2013; Cavalera et al., 2017).

Social and cultural norms may also contribute substantially to the observed gender differences, as gambling is often perceived—in Italy as in other European countries—as more socially acceptable for men, while women may face greater stigma when engaging in such behaviour (Baxter et al., 2016; Collard et al., 2022; McCormack et al. 2014; Rolando et al., 2023). Qualitative studies conducted in Italy further highlight how gambling practices have historically been embedded in male-dominated social settings in Southern Italy, thereby reinforcing their greater social legitimacy among men (Rolando & Beccaria, 2019).

Finally, gender roles and daily responsibilities may further contribute to this divide. Different studies show that family duties remain heavily unbalanced between genders, with women often carrying a greater share of caregiving and household responsibilities (Barigozzi et al., 2023; Hutt, 2020), leaving them with fewer opportunities—both in terms of time and accessibility—to participate in gambling activities.

Age, by itself, did not emerge as a significant predictor of regular gambling behaviour in the binary logistic regression analysis. This finding may reflect the interplay of several methodological and theoretical factors. First, older adults in the sample reported lower levels of perceived social approval of gambling from family and friends. Such disapproval may act as a social inhibitor, discouraging gambling engagement even amid lower psychosocial well-being. Moreover, individuals aged 60 and over may belong to generational cohorts less culturally familiar with, or inclined toward, gambling—particularly in its digital forms. As a result, gambling may be less normalized and less integrated into their daily lives. Additionally, older adults may experience reduced access to gambling opportunities due to technological barriers, financial limitations, or mobility constraints. These structural factors can further limit the likelihood of gambling, regardless of psychological vulnerability or motivational predispositions. Finally, it is important to consider that participants aged 60 and overrepresented only 28.7% of the total sample, which may have limited the statistical power to detect age-related effects.

### 5.6. Limitations

Several limitations should be considered when interpreting the findings of this study. First, due to the use of a convenience sampling method, the results are specific to the cultural and regional context examined and may not be generalizable, as the observed patterns could reflect sociocultural dynamics unique to Southern Italian communities. In addition, the demographic composition of the participating community organizations, characterized by a higher proportion of women and older adults, limits the representativeness of sex-stratified estimates. Accordingly, gender comparisons should be interpreted as within-sample patterns rather than population-level differences. Second, although we have primarily suggested that contextual factors such as psychosocial well-being and perceived social approval may account for gambling frequency, the cross-sectional design prevents us from establishing the temporal ordering of the observed associations. As discussed, it is also possible that gambling frequency influences levels of psychosocial well-being and perceived social approval. Longitudinal studies are therefore needed to clarify the reciprocal dynamics between psychosocial vulnerabilities, perceived norms, and gambling behaviour over time. Third, the reliance on self-reported data to assess gambling participation may introduce recall bias, potentially affecting the accuracy of participants’ responses—for example, by underestimating the prevalence of regular or at-risk gamblers. Fourth, the community-based nature of the sample leads to a predominance of non-gamblers and occasional gamblers, which inherently limits variability in gambling intensity. This skewed distribution may have reduced our ability to detect associations that emerge only at higher levels of gambling involvement. Future research would therefore benefit from recruiting participants not only in community settings but also in gambling-related contexts, allowing for a more comprehensive examination of risk and protective factors across groups characterized by different degrees of gambling involvement. It should also be noted that relying on gambling frequency during the past month ensured that respondents referred to recent gambling activity. However, this choice introduces some limitations: it does not allow us to distinguish lifetime non-gamblers from individuals who simply did not gamble during the reference month, nor does it allow us to determine whether higher levels of gambling frequency (e.g., monthly or weekly) reflect stable, long-term patterns or behaviour specific to that particular month. Finally, it is important to acknowledge that additional individual and psychosocial factors, such as physical and mental health, could mediate the observed relationships or provide alternative explanations, underscoring the need for future research to further elucidate the complex interplay of factors influencing gambling behaviour.

### 5.7. Implications for Practice

Although loneliness, low social support, and poor well-being are modifiable risk factors for gambling frequency, the literature on effective interventions to strengthen social connectivity and prevent non-problematic gamblers from becoming at-risk or problem gamblers is limited (Czaja et al., 2021). Recognizing problem gambling as a symptom of a deeper pathology of social bonds and coexistence, intrinsically linked to relational and social dynamics (M. Abbott et al., 2018; Messerlian et al., 2005; Reith et al., 2019; Stevens et al., 2021), we argue that its prevention requires sustained investment in the care and strengthening of interpersonal and community relationships. Gambling prevention programmes that help individuals identify alternative, more adaptive ways to connect with others and find need-supportive environments could help alleviate the social deficits that fuel at-risk gambling (Floyd et al., 2025). This perspective is particularly relevant considering contemporary societal changes—such as shifts in living arrangements, increased solitary living, shrinking family sizes, the decline of communal spaces like parks and post offices, and widespread digital technology use—that may make it increasingly difficult for individuals to access social support when facing life’s challenges. Strategies aimed at expanding social networks, strengthening social support, and promoting engagement in meaningful and enjoyable activities are likely to yield positive health outcomes. Such interventions could include the development of affordable, accessible programmes tailored to older adults, as well as initiatives that actively connect individuals to peer-support networks and community-based resources.

The action-research project “L’azzardo è fare rete” (“Gambling is Building Networks”)—within which the present study is situated—aimed to foster opportunities for dialogue and reflection within the involved communities. A series of awareness-raising meetings were held at the premises of partner organizations engaged in the research, targeting both service users and the wider public. While a detailed account of the intervention activities falls outside the scope of this paper, it is worth noting that these events were not only intended to present the findings of the exploratory research and critically discuss common myths about gambling, but also to encourage the sharing of personal experiences and to gather participants’ perspectives on the resources and challenges within their local contexts, in order to support local stakeholders in designing effective interventions.

## Figures and Tables

**Table 1:** Participants’ socio-demographic characteristics.

		Sex		Age		TOT	Chi-Square
		Women	Men	<60	≥60		
Educational level	No education or primary school	8 (1.3%)	8 (1.3%)	2 (0.3%)	14 (2.2%)	16 (2.5%)	
	Middle school	59 (9.3%)	41 (6.5%)	47 (7.4%)	53 (8.4%)	100 (15.8%)	χ^2^ sex = 9.795 (*p* < 0.05)
	High school	240 (37.9%)	89 (14.0%)	258 (40.7%)	71 (11.2%)	329 (51.9%)	χ^2^ age = 66.743 (*p* < 0.001)
	Bachelor’s degree or higher	131 (20.7%)	58 (9.1%)	145 (22.9%)	44 (6.9%)	189 (29.8%)	
Job status	Employee	139 (21.9%)	54 (8.5%)	158 (24.9%)	35 (5.5%)	193 (30.4%)	
	Freelancer	70 (11.0%)	15 (2.4%)	74 (11.7%)	11 (1.7%)	85 (13.4%)	
	Temporary worker	34 (5.4%)	42 (6.6%)	49 (7.7%)	27 (4.3%)	76 (12.0%)	χ^2^ sex = 64.249 (*p* < 0.001)
	Unemployed	99 (15.6%)	21 (3.3%)	108 (17.0%)	12 (1.9%)	120 (18.9%)	χ^2^ age = 299.801 (*p* < 0.001)
	Student	47 (7.4%)	52 (8.2%)	2 (0.3%)	97 (15.3%)	99 (15.6%)	
	Retired	49 (7.7%)	12 (1.9%)	61 (9.6%)	0 (0.0%)	61 (9.6%)	
Perceived income level	Not enough	56 (8.8%)	25 (3.9%)	54 (8.5%)	27 (4.3%)	81 (12.8%)	
	Cautious with expenses	112 (17.7%)	46 (7.3%)	116 (18.3%)	42 (6.6%)	158 (24.9%)	χ^2^ sex = 4.919 (*p* = 0.178)
	Enough to make ends meet	199 (31.4%)	79 (12.5%)	196 (30.9%)	82 (12.9%)	278 (43.8%)	χ^2^ age = 1.560 (*p* = 0.669)
	Comfortable	71 (11.2%)	46 (7.3%)	86 (13.6%)	31 (4.9%)	117 (18.5%)	

**Table 2:** Gambling habits, frequency and problem rates.

		Sex		Age		TOT	Chi-Square
		Women	Men	<60	≥60		
Gambling habits (*)	Lotteries (Scratch cards, Lotto, 10eLotto)	245 (68.4%)	113 (31.6%)	263 (73.5%)	95 (26.5%)	358 (56.5%)	χ^2^_sex_ = 0.162 (*p* = 0.687);
	*Adjusted residual*	−0.4	0.4	1.4	−1.4		χ^2^_age_ = 1.893 (*p* = 0.169)
	Slot machines and Bingo	36 (63.2%)	21 (36.8%)	42 (73.7%)	15 (26.3%)	57 (9.0%)	χ^2^_sex_ = 1.030 (*p* = 0.310)
	*Adjusted residual*	−1.0	1.0	0.4	−0.4		χ^2^_age_ = 0.175 (*p* = 0.676)
	Betting (Sports betting, Race betting, Private betting)	56 (42.7%)	75 (57.3%)	97 (74.0%)	34 (26.0%)	131 (20.7%)	χ^2^_sex_ = 53.626 (*p* < 0.001)
	*Adjusted residual*	−7.3	7.3	0.8	−0.8		χ^2^_age_ = 0.611 (*p* = 0.434)
	Blackjack and Online Poker	9 (50.0%)	9 (50.0%)	16 (88.9%)	2 (11.1%)	18 (2.8%)	χ^2^_sex_ = 3.160 (*p* = 0.075)
	*Adjusted residual*	−1.8	1.8	1.7	−1.7		χ^2^_age_ = 2.803 (*p* = 0.094)
Gambling frequency	No gambling	344 (78.5%)	105 (53.6%)	334 (73.9%)	115 (63.2%)	449 (70.8%)	
	*Adjusted residual*	6.4	−6.4	−0.3	0.3		
	Occasional gambling (**)	73 (67%)	36 (33%)	76 (69.7%)	33 (30.3%)	109 (17.2%)	χ^2^_sex_ = 73.295 (*p* < 0.001)
	*Adjusted residual*	−0.5	0.5	−0.4	0.4		χ^2^_age_ = 11.774 (*p* <.005)
	Regular gambling (***)	21 (4.8%)	55 (28.1%)	42 (9.3%)	34 (18.7%)	76 (12%)	
	*Adjusted residual*	−8.3	8.3	−3.3	3.3		
Severity Index	No problem gamblers	408 (93.2%)	145 (74.0%)	400 (88.5%)	153 (84.1%)	553 (87.2%)	
	*Adjusted residual*	6.7	−6.7	1.5	−1.5		
	Low risk gamblers	19 (4.3%)	26 (13.3%)	28 (6.2%)	17 (9.3%)	45 (7.1%)	
	*Adjusted residual*	−4.0	4.0	−1.4	1.4		χ^2^_sex_ = 46.013 (*p* < 0.001)
	Moderate risk gamblers	8 (1.8%)	17 (8.7%)	20 (4.4%)	5 (2.7%)	25 (3.9%)	χ^2^_age_ = 9.585 (*p* < 0.05)
	*Adjusted residual*	−4.1	4.1	1.0	−1.0		
	Problem gamblers	3 (0.7%)	8 (4.1%)	4 (0.9%)	7 (3.8%)	11 (1.7%)	
	*Adjusted residual*	−3.0	3.0	−2.6	2.6		

Adjusted residuals are reported for sex and age comparisons. Absolute values ≥ 1.96 indicate statistically significant deviations from expected counts. (*) Dummy variables; (**) Once a month; (***) More than once a month.

**Table 3:** Comparison of Target Variables by Sex and Age—Mann–Whitney U Test (Ranks).

Outcome	Group	N	Mean Rank	Sum of Ranks	Mann–Whitney U	*p*
Psychosocial Well-being Index	Men	196	292.99	57,425.50	38,119.50	0.024
	Women	438	328.47	143,869.50		
	<60	452	330.40	149,340.50	35,301.50	0.005
	≥60	182	285.46	51,954.50		
Perceived approval of gambling from family and friends	Men	196	344.98	67,616.50	37,537.50	<0.001
	Women	438	305.20	133,678.50		
	<60	452	324.11	146,498.00	38,144.00	0.034
	≥60	182	301.08	54,797.00		
Exposure to gambling by family and friends	Men	196	358.54	70,274.00	34,880.00	<0.001
	Women	438	299.13	131,021.00		
	<60	452	331.38	149,786.00	34,856.00	0.001
	≥60	182	283.02	51,509.00		
VOC1—Models of Engagement with the Social Context	Men	196	275.17	53,933.50	34,627.50	<0.001
	Women	438	336.44	147,361.50		
	<60	452	337.54	152,567.00	32,075.00	<0.001
	≥60	182	267.74	48,728.00		
VOC2—Ways of Evaluating the Social Context	Men	196	309.24	60,610.50	41,304.50	0.447
	Women	438	321.20	140,684.50		
	<60	452	317.06	143,311.50	40,933.50	0.924
	≥60	182	318.59	57,983.50		

Note. VOC1 = Models of Engagement with the Social Context (Radical Distrust to Moderate Trust). VOC2 = Ways of Evaluating the Context (Moderate Criticism to Idealization). Mann–Whitney U tests compare distributions across groups; *p*-values two-tailed.

**Table 4:** Binary logistic regression results predicting likelihood of regular gambling.

Predictor	B	SE	Wald z	*p*	OR	95% CI for OR
Intercept	−1.40	0.20	−7.05	<0.001	—	—
Psychosocial Well-being Index	−0.34	0.14	−2.43	<0.05	0.71	[0.54, 0.94]
Perceived social approval	0.46	0.13	3.57	<0.001	1.58	[1.23, 2.04]
Exposure to gambling	0.20	0.16	1.27	0.204	1.22	[0.89, 1.67]
Sex (Women)	−1.85	0.30	−6.20	<0.001	0.16	[0.09, 0.28]
Age	0.24	0.16	1.53	0.127	1.27	[0.94, 1.72]
VOC1 *	0.04	0.38	0.11	0.916	1.04	[0.49, 2.19]
VOC2 **	1.08	0.41	2.66	<0.01	2.94	[1.31, 6.49]
VOC1 × Age	−0.01	0.01	−0.87	0.385	0.99	[0.98, 1.01]
VOC2 × Age	−0.02	0.01	−2.54	<0.05	0.98	[0.96, 0.99]

* VOC 1: Models of Engagement with the Social Context: Radical Distrust (−) vs. Moderate Trust (+); ** VOC2—Ways of Evaluating the Context: Moderate Criticism vs. Idealization.

## Data Availability

The data presented in this study are available on request from the corresponding author due to ethical restrictions related to participant consent.

## Appendix A

**Table A1:** Response modes most significantly associated with the first factorial dimension (VOC1) of the View of Context questionnaire.

Absolute Distrust (−)			Moderate Trust (+)		
*Variable*	*Modality*	*Test Value **	*Variable*	*Modality*	*Test Value **
Nowadays people don’t know who they can count on	Strongly agree	−14.18	Reliability: Government	Quite reliable	10.71
It’s pointless to turn to those in public office because these people are often not truly interested in the problems of the people.	Strongly agree	−14.04	Sometimes it is better to keep quiet so as not to have problems	Quite disagree	10.27
Nowadays people are forced to live day	Strongly agree	−13.12	Reliability: Public Administration	Quite reliable	10.16
Reliability: Government	Not reliable at all	−12.22	It’s pointless to turn to those in public office because these people are often not truly interested in the problems of the people.	Quite disagree	9.95
Sometimes it is better to keep quiet so as not to have problems	Strongly agree	−12.05	Reliability: Healthcare	Quite reliable	9.30
To be successful in life. you need to have the right knowledge	Strongly agree	−11.82	Nowadays people don’t know who they can count on	Quite disagree	9.04
Sometimes it is necessary to break the rules to help loved ones	Strongly agree	−11.75	To be successful in life. you need to have the right knowledge	Quite disagree	8.84
Reliability: Healthcare	Not reliable at all	−11.59	Reliability: Law enforcement	Quite reliable	8.54
It is not possible to make predictions about the future	Strongly agree	−11.05	Reliability: Church	Quite reliable	8.48
Reliability: Public Administration	Not reliable at all	−10.22	Reliability: School	Quite reliable	8.36
People are not capable of change	Strongly agree	−10.22	Think about the coming years. Your future will be	Slightly improved	7.94
It is useless to worry. as it is not possible to influence what happens	Strongly agree	−9.66	Nowadays people are forced to live day	Quite disagree	7.71
Those who achieve success in life must thank luck	Strongly agree	−9.23	To succeed in life is important: [siding with the strongest]	Not very much	7.64
To succeed in life is important: [siding with the strongest]	Very much	−8.74	Imagine the place where you live five years from now. How will you feel living there?	Better	7.61
Reliability: Public Transport	Not reliable at all	−8.44	It is useless to worry. as it is not possible to influence what happens	Quite disagree	7.33
Reliability: Law enforcement	Not reliable at all	−8.40	It is not possible to make predictions about the future	Quite disagree	7.11
To succeed in life is important: [having few scruples]	Very much	−7.69	Sometimes it is necessary to break the rules to help loved ones	Quite disagree	7.09
Reliability: School	Not reliable at all	−7.22	Those who achieve success in life must thank luck	Quite disagree	6.91
Imagine the place where you live five years from now. How will you feel living there?	Much worse	−7.19	Reliability: Public transport	Quite reliable	5.97
To succeed in life is important: [sharing]	Not at all	−6.93	To succeed in life is important: [understanding the world]	Quite a bit	5.66

* Highest levels of associations standard scores.

**Table A2:** Response modes most significantly associated with the second factorial dimension (VOC2) of the View of Context questionnaire.

Moderate Criticizing (−)			Idealizing (+)		
Variable	Modality	Test Value *	Variable	Modality	Test Value *
Sometimes it is better to keep quiet so as not to have problems	Quite agree	−10.43	It is useless to worry. as it is not possible to influence what happens	Strongly disagree	14.30
Nowadays people don’t know who they can count on	Quite agree	−9.75	To be successful in life. you need to have the right knowledge	Strongly disagree	14.01
To be successful in life. you need to have the right knowledge	Quite agree	−9.53	It is not possible to make predictions about the future	Strongly disagree	13.43
It’s pointless to turn to those in public office because these people are often not truly interested in the problems of the people	Quite agree	−9.03	People are not capable of change	Strongly disagree	12.96
It is not possible to make predictions about the future	Quite agree	−9.02	Those who achieve success in life must thank luck	Strongly disagree	12.32
To succeed in life is important: [sharing]	Quite a bit	−8.95	Nowadays people are forced to live day	Strongly disagree	12.20
To succeed in life is important: [siding with the strongest]	Quite a bit	−8.08	Sometimes it is necessary to break the rules to help loved ones	Strongly disagree	11.88
It is useless to worry. as it is not possible to influence what happens	Quite disagree	−8.02	Nowadays people don’t know who they can count on	Strongly disagree	11.64
Sometimes it is necessary to break the rules to help loved ones	Quite agree	−8.00	Sometimes it is better to keep quiet so as not to have problems	Strongly disagree	11.63
People are not capable of change	Quite agree	−7.87	To succeed in life is important: [siding with the strongest]	Not at all	10.90
To succeed in life is important: [having few scruples]	Quite a bit	−7.26	To succeed in life is important¨: [sharing]	Very much	10.58
To succeed in life is important: [following rules]	Quite a bit	−7.22	It is useless to turn to those in public office, because they are often not truly interested in people’s problems.	Strongly disagree	9.97
Nowadays people are forced to live day	Quite agree	−7.07	To succeed in life is important¨: [having few scruples]	Not at all	9.53
Those who achieve success in life must thank luck	Quite agree	−7.04	To succeed in life is important: [following rules]	Very much	9.12
To succeed in life is important: [Conforming to prevailing trends]	Quite a bit	−6.79	Imagine the place where you live five years from now. How will you feel living there?	Much better	8.02
It is useless to worry. as it is not possible to influence what happens	Quite agree	−6.64	Reliability: Law enforcement	Very reliable	7.96
Reliability: Government	Not very reliable	−6.59	Reliability: School	Very reliable	7.92
Those who achieve success in life must thank luck	Quite disagree	−6.56	Think about the coming years. Your future will be	Improved	7.86
Reliability: Public Administration	Not very reliable	−6.33	Reliability: Church	Very reliable	7.85
To succeed in life is important¨: [to acquire knowledge]	Quite a bit	−6.22	Reliability: Public Administration	Very reliable	7.81
People are not capable of change	Quite disagree	−5.04	Reliability: Government	Very reliable	7.16
Reliability: public transport	Not very reliable	−5.03	To succeed in life is important: [Conforming to prevailing trends]	Not at all	5.34
Imagine the place where you live five years from now. How will you feel living there?	Worse	−4.82	Reliability: public transport	Very reliable	4.74
Reliability: Church	Not very reliable	−4.42	To succeed in life is important: [to acquire knowledge]	Very much	4.48
Reliability: Law enforcement	Not very reliable	−4.28	Reliability: Health system	Very reliable	4.13
Reliability: Health system	Not very reliable	−4.00	To succeed in life is important: [Conforming to prevailing trends]	Not very much	3.82
To succeed in life is important: [to understand the world]	Quite a bit	−3.96	Reliability: Public transport	Quite reliable	3.39
Think about the coming years. Your future will be	Slightly improved	−3.94	To succeed in life is important: [following rules]	Not at all	3.32
To succeed in life is important: [following rules]	Not very much	−3.85	To succeed in life is important: [to understand the world]	Very much	3.15

* Highest levels of associations standard scores.

**Table A3:** Pearson correlations between psychosocial variables and gambling frequency.

		LS	ELS	SLS	SS	FS	PSI	LDS1	LDS2	Age	GA	GE
Gambling frequency	Pearson Correlation	0.031	−0.050	0.105 **	−0.133 **	−0.092 *	−0.138 **	−0.030	−0.189 **	0.113 **	0.223 **	0.216 **
	Sig. (2-tailed)	0.436	0.207	0.008	0.001	0.020	0.000	0.444	0.000	0.004	0.000	0.000
	N	634	634	634	634	634	634	634	634	634	634	634

**. Correlation is significant at the 0.01 level (2-tailed). *. Correlation is significant at the 0.05 level (2-tailed). LS—Loneliness Scale; ELS—Emotional loneliness Scale; SLS—Social loneliness Scale; SS—Social support; FS—Flourishing Scale; PSI—Psychosocial Well-being Index; GA—Gambling approval; GE—Gambling exposure.
